# The WHO UNESCO FIP Pharmacy Education Taskforce

**DOI:** 10.1186/1478-4491-7-45

**Published:** 2009-06-05

**Authors:** Claire Anderson, Ian Bates, Diane Beck, Tina Penick Brock, Billy Futter, Hugo Mercer, Mike Rouse, Sarah Whitmarsh, Tana Wuliji, Akemi Yonemura

**Affiliations:** 1 School of Pharmacy, University of Nottingham, Nottingham, UK; 2 The School of Pharmacy, University of London, London, UK; 3 College of Pharmacy, University of Florida, Gainesville, Florida, USA; 4 Capacity Building & Performance Improvement, Management Sciences for Health, Washington DC, USA; 5 Faculty of Pharmacy, Rhodes University, Grahamstown, South Africa; 6 Health Workforce Education and Production, Department of Human Resources for Health, World Health Organization, Geneva, Switzerland; 7 Accreditation Council for Pharmacy Education, Chicago, Illinois, USA; 8 Pharmacy Education Taskforce, London, UK; 9 International Pharmaceutical Federation, The Hague, the Netherlands; 10 Section for Reform, Innovation and Quality Assurance, Division of Higher Education, United Nations Educational, Scientific and Cultural Organization, Paris, France

## Abstract

Pharmacists' roles are evolving from that of compounders and dispensers of medicines to that of experts on medicines within multidisciplinary health care teams. In the developing country context, the pharmacy is often the most accessible or even the sole point of access to health care advice and services.

Because of their knowledge of medicines and clinical therapeutics, pharmacists are suitably placed for task shifting in health care and could be further trained to undertake functions such as clinical management and laboratory diagnostics. Indeed, pharmacists have been shown to be willing, competent, and cost-effective providers of what the professional literature calls "pharmaceutical care interventions"; however, internationally, there is an underuse of pharmacists for patient care and public health efforts. A coordinated and multifaceted effort to advance workforce planning, training and education is needed in order to prepare an adequate number of well-trained pharmacists for such roles.

Acknowledging that health care needs can vary across geography and culture, an international group of key stakeholders in pharmacy education and global health has reached unanimous agreement that pharmacy education must be quality-driven and directed towards societal health care needs, the services required to meet those needs, the competences necessary to provide these services and the education needed to ensure those competences. Using that framework, this commentary describes the Pharmacy Education Taskforce of the World Health Organization, United Nations Educational, Scientific and Cultural Organization and the International Pharmaceutical Federation Global Pharmacy and the Education Action Plan 2008–2010, including the foundation, domains, objectives and outcome measures, and includes several examples of current activities within this scope.

## Introduction

Access to essential medicines is one of the most basic health services. To ensure access to and appropriate use of medicines, there is a need for an appropriately-trained pharmacy workforce. Unfortunately, pharmacists and pharmacy support personnel in many countries are too few in number and trained at a critically insufficient scale. Pharmacists represent the third largest health care professional group in the world after nurses and doctors. The ratio of the pharmacy workforce to population varies widely between countries, from 0.8 per 10 000 population in the African region to 5.4 in the Americas [1]. For example, at present there is one pharmacist for every 1300 people in the United Kingdom, but in Uganda, there is only one pharmacist for every 140 000 people, and local health authorities estimate that this figure represents only one third of the required pharmacist workforce. The scaling up and quality improvement of pharmacy education and training are essential for addressing workforce shortages and for meeting basic health needs.

The international shortage of health care professionals exists in different severities and has different root causes, depending on the particular health profession and the country of origin. In addition, even with adequate numbers of health care providers, because health care priorities differ between countries, one universal health workforce model would invariably not provide services tailored efficiently to all those who need them. Still, due to the increasing overlap of professional roles and team-based clinical services, it is essential that countries consider all health professionals, including pharmacists and pharmacy support personnel, when developing workforce plans [2].

### Appropriate use of medicines

The United Nations Working Group on Access to Essential Medicines has advised that any attempts to strengthen the health system to improve access to and appropriate use of medicines will be undermined without tackling the underlying pharmacy workforce shortages and imbalances [1]. This is advice that some countries are adopting, albeit slowly.

In case reports from across the globe, pharmacists and pharmacy support personnel have been shown to be willing, competent and cost-effective providers of patient-focused and medicines-centered care (termed "pharmaceutical care" in the professional literature) to individuals and populations. Pharmacists' roles are evolving from that of compounders and dispensers of medicines to that of medicines experts within multidisciplinary health care teams. In the developing-country context, the pharmacy is often the most accessible or even the sole point of access to health care advice and services.

Because of their knowledge of medicines and clinical therapeutics, pharmacists are suitably placed for task shifting in health care and could be further trained to undertake functions such as clinical management and laboratory diagnostics. Pharmacy personnel are also ideally placed for public health roles, although this function remains largely untapped. Indeed, the underuse of the pharmacy workforce for preventive and treatment-based roles is widely acknowledged [3-6]. To improve health outcomes, a coordinated and multifaceted effort to advance workforce planning, training and education is needed.

### Defining pharmacy education

For the intents and purposes of the Taskforce, when using the term pharmacy education, it is to be understood that this refers to the educational design and capacity to develop the workforce for a diversity of settings (e.g. community, hospital, research and development, academia) across varying levels of service provision and competence (e.g. technical support staff, pharmacists and pharmaceutical scientists) and scope of education (e.g. undergraduate, postgraduate, lifelong learning). This multidimensional conceptualization embodies a systematic approach to education development that enables and supports a sustainable expert health care workforce to effectively improve health.

### Needs-based education

Needs-based education is a strategy that calls for any given system to assess the needs of its community and then develop (or adapt) the supporting educational system accordingly. Health care demands are incredibly diverse and complex, often varying widely within and between regions. Although broad, general frameworks may be beneficial at the macro level; "one-size-fits-all" systems do not offer the authenticity needed for buy-in and sustainability at the micro level.

To date, much of the focus on developing the academic workforce and then practitioners has involved bringing academics to the developed world for research (PhD) or practice (MSc in clinical pharmacy or PharmD) training in institutions of higher education. There has been less concentration on developing teachers who can significantly increase the throughput of high-quality trained pharmacists for the workforce.

The United States-Thai Consortium and the Thai-United Kingdom Collaborative Research Network each represent ongoing examples of partnership for needs-based training focused on building capacity in pharmaceutical services and sciences in Thailand. These programmes, which include collaborations among 10 Thai schools, 10 United States schools and 11 United Kingdom schools, allow for Thai pharmacy students, pharmacy practitioners and scientists to undertake government-subsidized advanced pharmacy studies (e.g. clinical and doctoral level) in the United States and United Kingdom to build capacity for the academic workforce.

Since inception of these programmes (1993 in the United States and 2003 in the United Kingdom), approximately 200 Thai practitioners and researchers have completed studies in the United States and United Kingdom, returning to Thailand as clinicians, educators and researchers. Continuing annual consultations and "reverse exchanges" have ensured that the programme is refined and adapted to remain authentic.

Additional examples of anchoring pharmacy education to local needs are two sites in Kenya. The Purdue Kenya Programme is long-running and has trained more than 50 United States pharmacy students and residents at a hospital associated with Moi University in Eldoret. Last year the programme began pairing United States students (Purdue University School of Pharmacy) with Kenyan pharmacy students (University of Nairobi School of Pharmacy) to provide clinical pharmacy services in the hospital.

These student pairs are actively involved with developing new pharmacy-managed clinics in areas such as diabetes and anticoagulation services. The United States students mentor the Kenyans with regard to team-based approaches to improving care; the Kenyan students mentor the Americans regarding the culture and mechanisms for providing such care in that region.

Pharmacists at Aga Khan University Hospital in Nairobi are developing a part-time, work-based postgraduate diploma in clinical pharmacy to be offered to pharmacists throughout the Nairobi area. These pharmacists will then be able to educate other local pharmacists and pharmacy students. Because this programme works with the local medical and nursing community, it is conjectured that the enhanced pharmacy services will not be seen as a threat to existing services, but rather as a complement to them to enable a team-based approach to health care services.

One of the crucial needs, particularly in developing countries, is to train pharmacists who have internalized their role of helping to meet the medicine-related needs of poorer, less urbanized communities. Few students are familiar with these settings prior to training and most students appear to aspire to work in well-equipped tertiary hospitals in cities.

Developing a commitment to stay and service these needs is more likely to occur when the students spend time in the social laboratory provided at primary health care settings and in patient's homes. Many innovative education practices have evolved to fill this gap. For example, in South Africa, Rhodes University staff members supervise pharmacy student visits to patients in their homes. Students are briefed on the patients' details in advance, and supported by interpreters. Their role is primarily to detect medicine-related problems, provide education where this is appropriate and refer if necessary. This innovative programme received the university's first Vice Chancellor's Community Engagement Award in January 2008 as a testimony of the success of its capacity to address the needs of a community through experiential learning.

All these examples represent the adaptation of general educational strategies such as partnering between schools, seeding research leaders, investment in a train-the-trainers programme, expanding the clinical portion of the professional curriculum and engaging communities. Each strategy has been flexible to the pre-existing and future needs of the community in order to optimize effectiveness. This further supports the importance of the adoption of a vision and action plan for global pharmacy that is founded in local, regional, national and international needs for health care.

### The pharmacy workforce

The scaling up and quality improvement of pharmacy education and training are essential for tackling workforce shortages, meeting basic health needs and saving lives. They form one of the major bottlenecks in expanding the pharmacy workforce. The capacity to provide pharmaceutical services in each country depends on two workforce needs: an appropriately trained pharmacy workforce to provide the services and a competent and committed academic workforce to train sufficient numbers of new pharmacists and other pharmacy support staff at both basic and enhanced levels. Each depends on appropriately resourced academic institutions composed of students who have the necessary intellectual knowledge, values and competence to be change agents for health in their communities. We also anticipate a further demand for academic pharmacists as continuing professional development requirements increase for qualified pharmacists.

One response to the global shortage of pharmacists has been an increase in the size and number of pharmacy schools in both developed and developing countries. An expansion in the number of pharmacy graduates occurred or was recommended in Australia, Canada, Ireland, Northern Ireland, the United Kingdom and the United States [7-11]. There have also been large increases in China and India.

However, the global data on pharmacy schools are far from complete. There has been an increase in the number of pharmacy schools and increases in enrolments at existing schools. This strategy has been successful in increasing the hospital pharmacist workforce in Australia, Ghana and the United Kingdom. Additionally there may be difficulties at first in ensuring a sufficient number of pre-registration or residency training posts for an increased number of graduates seeking to enter the workforce [12,13]. In many countries, workforce shortages also apply to academia; capacity to scale up education may therefore be limited.

Expansion presents many concerns, including its effect on the quality of teaching, the number of available pharmacy-trained academic faculty members and the academic standard of applicants. Higher education funding policies have often encouraged higher enrolments, which have not been matched by similar increases in resources, including staffing levels [8].

Alignment of curricula with actual practice activities is important for a number of reasons, including job satisfaction and to provide the best health care for patients. Matowe et al. [14] point out that pharmaceutical practice differs widely from what students were taught at university.

Another misalignment of pharmacy education, highlighted in developing countries, is that pharmacy schools are largely located in urban areas; therefore, the majority of students are from relatively near the urban centres. This fact, alongside the fact that the pharmacy curriculum is similar to that of more developed countries, meant that graduates have little relevant understanding and skills required for addressing health problems in rural areas of their own country; administrators realize that their ambitions are unlikely to be met in these rural locations [15]. A perceived lack of educational and professional opportunities available to pharmacists in Ghana was seen as preventing them from making a full contribution to health care in Ghana [16].

Recognizing the need to develop a vision for pharmacy education, ensure a sustainable pharmacy workforce relevant to needs and build the local capacity of pharmacy higher-education institutions, the International Pharmaceutical Federation (FIP) launched the Pharmacy Education Taskforce with the World Health Organization (WHO) and the United Nations Educational, Scientific and Cultural Organization (UNESCO) in March 2008 after a series of global consultations on pharmacy education. The Taskforce is a collection of stakeholders representing various global, regional and country networks with the shared goal of coordinating and catalysing actions to develop pharmacy education. The purpose of the Taskforce is to oversee the implementation of the 2008–2010 Pharmacy Education Action Plan, identify resources and serve as a connection for stakeholders [17].

### Pharmacy Education Action Plan 2008–2010

Acknowledging that specific health care needs can vary significantly across geography and culture, an international group of key stakeholders has reached unanimous agreement that pharmacy education must be quality-driven and directed towards these identified needs, the pharmaceutical services needed to meet these needs, the competences needed to provide these pharmaceutical services and the education required to achieve/ensure these competences. The action plan aims to: develop a vision, frameworks, guidelines and case studies; build evidence and advocacy; accelerate country action; and establish a global platform for dialogue [18]. The Action Plan is dedicated to four domains of action: quality assurance, academic and institutional capacity, and competence and vision for pharmacy education. Each domain of action represents a work stream that is phased over the three years to include country case studies, consensus building and policy guidance. The focus of these case studies is the sub-Saharan African region, due to the urgency of the health workforce crisis and extreme pharmacy workforce shortages.

**Figure 1:**
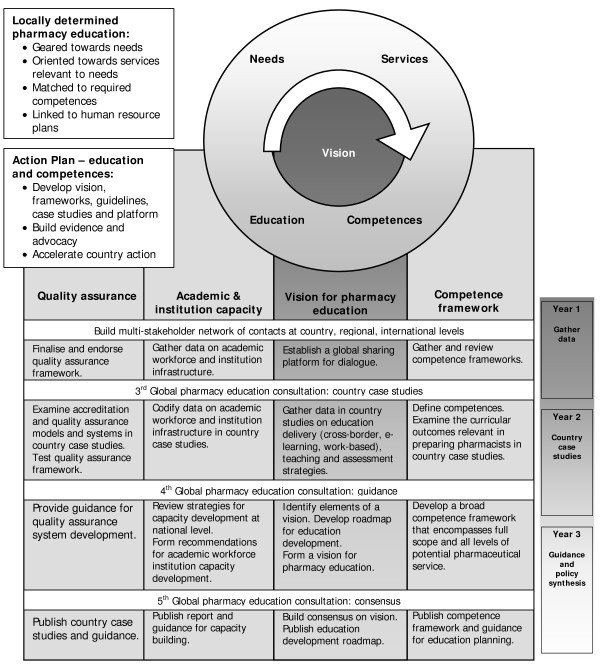
**Pharmacy Education Action Plan 2008 – 2010**.

The Pharmacy Education Action Plan was developed and refined during two global pharmacy education consultations convened by FIP [19,20]. It will be actively and continuously monitored by the Taskforce to assess progress towards the overreaching goal; i.e. disseminating evidence-based guidance and frameworks that facilitate the development of pharmacy education (and higher education capacity) to enable sustainability of a pharmacy workforce appropriately skilled to provide pharmaceutical services. The Global Pharmacy Education Action Plan 2008–2010 represents the greatest opportunity to date for stakeholders to support, participate, contribute towards and commit to action for pharmacy education. Figure 1 depicts the Action Plan, a vision for pharmacy education based on developing competent pharmacists to providing services based on local needs, goals and outcomes in four priority domains: quality assurance, academic and institutional capacity, vision for pharmacy education and competence, for each year of the project.

### Pharmacy Education Action Plan work streams

#### Quality assurance

The quality assurance project team is continuing and advancing the work of the FIP International Forum for Quality Assurance of Pharmacy Education. This Forum has collected and examined (national) quality standards and systems that reflect contemporary pharmaceutical services and meet the needs of the specific country for which they were developed. Similar to work of the vision and competence project team, these systems have been examined to identify the principles and core elements of quality assurance that are unlikely to vary by culture.

From this common ground, an internationally acceptable quality assurance framework has been proposed and the first round validation/testing was done via two country case studies (in Ghana and Zambia). The next round will involve around 20 countries via an online survey instrument. Four persons from different but relevant perspectives will be targeted to participate from each country.

The project team is working actively with WHO, and has completed the first draft of a self-assessment instrument for pharmacy (pharmacist) education. The instrument uses the format (Structure, Process, Outcomes) and quality criteria of the Global Framework and was adapted from a generic self-assessment instrument developed by WHO, based on the WFME Global Standards [21]. The draft instrument is undergoing its first round of review and will then go through a validation/testing phase via country case studies.

#### Academic and institutional capacity

The academic and institutional capacity project team is targeting its work on issues well documented in the medical literature relating to the sustainable development of the academic workforce, such as disincentives towards careers in pharmacy academia; absence of clear career pathways, particularly for clinical teachers; and a culture that often prioritizes grants and peer-reviewed publications over effective teaching efforts [22]. The team is also exploring cases where poor physical infrastructure (e.g. safety concerns, absence of even basic facilities, resources and physical capacity) serve as primary barriers to good education and capacity building.

An in-depth case study in one African country will investigate barriers and facilitators to capacity building in pharmacy education; define roles and responsibilities of pharmacists in enhancing health in African countries; and attempt to synthesize innovative strategies to recruiting and developing the academic workforce We will examine all the issues with a wide variety of stakeholders at ministry, university and practice levels with a view to using this qualitative data to produce a survey instrument to use with a number of other countries.

From this work and an analysis of academic workforce development strategies, evidence-based guidance for the academic and institutional capacity development will be generated. The Taskforce is also working with WHO and the University of Copenhagen on the Avicenna Global Directories of Education Institutions for Health Professions, a publicly accessible database of schools, colleges, and universities for education of academic professions in health, including pharmacy [23].

#### Vision and competence

The vision and competence project team is developing an "educational roadmap" to guide efforts in and mechanisms for pharmacy education. Countries, particularly those marginalized by the human resources for health crisis, can use this evidence to develop their workforce and to track the results of their efforts. This domain of work is examining existing competence frameworks and use of these before initiating a consultative and evidence-based process to develop a broad competence framework for the pharmacy workforce.

As part of this process, the relationship between culture of competency (and any perceptions therein) is being explored. A workshop at the International Social Pharmacy Workshop in New Zealand (July 2008) first attempted to explore these ideas, and it is clear that no one particular competence model will meet the needs of all parties. However, identifying the core tenets that support all pharmaceutical services along the continuum from research to public health and allow for a grounded foundation and framework with flexibility for adaptation (based on local needs) is a key principle.

#### UNITWIN Network

One strategic approach that has been adopted by the Taskforce has been to form a partnership with UNESCO, with the aim of using the experience of a global agency and coupling this directly to the pharmacy higher education sector across regions. The designated Global Pharmacy Education Development Network UNITWIN platform will act as a conduit for developing consensus and facilitating the spread of best practice and educational development worldwide. The UNITWIN Network will establish a resource base and collaborative forum for exchange, research and capacity building dedicated to tackling challenges of academic capacity, quality assurance of educational systems and workforce competence. This is the first time that a formal global network has been established for pharmacy education under the stewardship of the professional body and United Nations agencies.

The capacity to provide relevant pharmaceutical services in each country depends on an appropriately trained workforce to provide these services and a competent and committed academic workforce to provide education and training at all levels.

#### Community of Practice

The Taskforce has also formed a Community of Practice (CoP), an online global platform where Taskforce members can view and post documents and resources, take part in discussions and keep informed of events and activities. The Taskforce CoP currently connects more than 200 people from 56 different countries.

## Conclusion

The WHO UNESCO FIP Pharmacy Education Taskforce provides a conduit and mechanism for collective global action. The Taskforce objectives are to develop a vision for pharmacy education; advocate the development of a sustainable pharmacy workforce relevant to needs (health, education and market); investigate the limited capacity of pharmacy higher education institutions, particularly in developing countries; and provide a framework for quality assurance of pharmacy education. In going beyond the rhetoric of needs-based education, 2008–2010 marks the roll-out of the Global Pharmacy Education Action Plan and thus field testing for learning and sharing to harvest collective results that spur education development.

## Competing interests

The authors declare that they have no competing interests.

## Authors' contributions

All the authors (CA, IB, DB, TPB, BF, HM, MR, SW, TW, AY) made substantial contributions to the conception, design and drafting of this paper and they approve the publication of this version.

## Authors' information

All the authors are members of the Pharmacy Education Taskforce. CA is a member of the Board of Pharmacy Practice, International Pharmaceutical Federation, and Professor of Social Pharmacy, University of Nottingham. IB is Vice-President of the European Association of Faculties of Pharmacy and Professor and Head of Education, The School of Pharmacy, University of London. DB is a Past President of the American Association of Colleges of Pharmacy and Professor and Director of Educational Initiatives, College of Pharmacy, University of Florida. TPB is the Director, Capacity Building & Performance Improvement, Management Sciences for Health. BF is an Associate Professor of the Faculty of Pharmacy, Rhodes University. HM is Acting Coordinator of Health Workforce Education and Production, Department of Human Resources for Health, World Health Organization, Geneva. MR is the Convener of the International Forum of Quality Assurance in Pharmacy Education, Academic Section, International Pharmaceutical Federation, and a member of the Accreditation Council for Pharmacy Education ACPE). SW is the Communications Coordinator of the Pharmacy Education Taskforce, International Pharmaceutical Federation. TW is a Project Manager for the International Pharmaceutical Federation. AY is a Programme Specialist, Section for Reform, Innovation and Quality Assurance; Division of Higher Education; United Nations Educational, Scientific and Cultural Organization.
